# Disordered eating symptoms in Austrian men of different ages in the context of fitness centers

**DOI:** 10.1007/s40519-021-01317-y

**Published:** 2021-11-02

**Authors:** Barbara Mangweth-Matzek, Benjamin Decker, Irene Erschbaumer, Verena Wurnig, Georg Kemmler, Carina S. Bichler, Claudia I. Rupp

**Affiliations:** 1 Department of Psychotherapy, Psychotherapy and Psychosomatics, University Hospital of Psychiatry II, Medical University Innsbruck, Innsbruck, Austria; 2 Department of Sport Science, University of Innsbruck, Innsbruck, Austria; 3 Department of Psychotherapy, Psychotherapy and Psychosomatics, University Hospital of Psychiatry I, Medical University Innsbruck, Innsbruck, Austria

**Keywords:** Fitness center, Men, Eating disorder, Body image, Excessive exercise

## Abstract

**Purpose:**

To assess eating behavior and associated factors in male fitness-center attendees.

**Methods:**

An anonymous questionnaire was administered to male fitness center members of Innsbruck (Austria), aged 18–80 years to assess socio-demographic features, weight history, sports activity, eating behavior including disordered eating based on the Eating Disorder Examination Questionnaire (EDE-Q) and DSM-5 key symptoms for eating disorders (anorexia nervosa, binge eating, bulimia nervosa, purging disorder) and body image. Three age groups (younger—middle-aged—older men) were compared regarding the variables described above.

**Results:**

A total of 307 men included displayed high rates of disordered eating as described by EDE-Q cutoff scores (5–11%) as well as by DSM-5 eating disorder symptoms (10%). While EDE-Q cutoff scores did not differentiate between the groups, the prevalences of DSM-5 eating disorder symptoms yielded significant differences indicating a clear decrease with increasing age. Binge eating and bulimic symptoms with excessive exercising as the purging method were the most often reported symptoms.

**Conclusion:**

Although described as typically female, disordered eating does occur in male fitness-gym attendees across all ages. The older the men, the less prevalent are the symptoms. Awareness of disordered eating and possible negative effects need to be addressed for attendees and trainers of the gym.

**Level of evidence:**

V—descriptive survey study.

## Introduction

Preoccupation with beauty, fitness, and physical appearance in Western countries has led to increasing sports activities including exercising in the gym. For over half a century, slim body size is still the main ideal for the majority of women, it appears that a muscular body has become the ideal for a huge proportion of men next to fitness and leanness [1].

Although sports and exercise are the means to get or stay healthy, there is evidence of unhealthy consequences due to “overdosing sports” and lacking recovery [2]. Although not part of existing diagnostic classifications, “exercise addiction” describes a behavior that is researched quite intensively in the last years [3, 4].

Less known is that DSM-5 criteria of bulimia nervosa include “excessive sports activity” as a possible purging method in the context of bulimia nervosa describing compensation for binge eating and a method to prevent weight gain [5, 6]. However, there is little data on this particular aspect, since most affected bulimic persons use self-induced vomiting or laxatives as a means to compensate against weight gain after binge eating [5].

There is scientific evidence that disordered eating is more prevalent among athletes than in the general population [7] based on the concept of “female athlete triad syndrome” first described in 1992 by the American College of Sports Medicine [8]. Although women are yet the predominant gender in eating disorders, men are also affected, but still “underdiagnosed, undertreated, and misunderstood” [9]. Next to those men who feel too fat and start to be anorexic or bulimic, there are men, who are described to become eating disordered driven by an ideal of Adonis-like muscularity [10–12]. The fitness centers as institutions of “health promotion” seem to be associated with an acceptance regarding “disordered eating behavior”. This suggests that within the sports world, deviations of normal eating behavior are less likely stigmatized [10].

From this point of view, it has to be considered that athletic workout in the gym can be instrumentalized in the context of disordered eating behavior. It is of interest that to the best of our knowledge, there are only a few studies on fitness-center attendees and disordered eating and none regarding men of different ages in that context. Müller et al. [13] studied various forms of “addictive behaviors” among 128 fitness center clients aged on average 27 years (72% male) and reported 11% showing eating disorder pathology based on the EDE-Q (8% among the men). Stapleton et al. [1] compared male gym users (*N* = 140, aged 18–65 years) and non-gym users (*N* = 40, aged 18–63 years) regarding body image avoidance, body dissatisfaction and eating pathology and found significant differences between the two groups. Gym users endorsed higher body dissatisfaction and were engaged more in disordered eating than non-gym users. Lichtenstein et al. [14] reported that “compulsive exercise” might be a socially accepted behavior in fitness centers, and described a linkage to eating disorder pathology, emotional problems and personality traits such as perfectionism, neuroticism or narcissism. There are a few studies that focus on fitness instructors’ and employee’s recognition or suspicion of disordered eating and excessive exercise in participants [15–17]. This research suggests that there is a high level of insecurity in identifying and managing the problems, to differentiate between healthy and unhealthy behavior and many open questions how to address the issues interpersonally.

In the context of Austrian data and eating disorders in males, we have published a community study in 2016 [18] identifying 32 (7%) participants, aged 40–75 years, meeting criteria for eating disorder symptoms as defined by DSM-IV as compared to 438 (93%) men with normal eating.

In summary, research in males with disordered eating is still very marginal, although new characteristics have been described [9, 11].

The aim of this study was to examine eating behavior (including disordered eating assessed by the EDE-Q and by DSM-5 key symptoms of eating disorders), sports activity, and body image in male fitness-center attendees of different age groups (younger —middle-aged—older men).

## Method

### Study design and participants

We distributed our anonymous questionnaires to 652 male participants of the two largest fitness centers in Innsbruck, Austria, aged 18–80 years. Interested men were administered the questionnaire including the informed consent and were asked to return both completed forms into a sealed box positioned in the corner of the fitness center’s entrance. We recruited men of all ages and combined two decades of age to form three groups including younger (18–40 years), middle-aged (41–60 years) and older men (61–80). 307 subjects returned and completed the questionnaire and were included in our statistical analysis (yielding a participation rate of 45.7%). Since two men did not report their age, we did the statistical analysis regarding the three age groups based on 305 men. We do not have explicit information about those men who did not return the questionnaire.

All study procedures were approved by the Ethics Commission of the Medical University of Innsbruck, Austria.

### Study instrument

To maximize the participation rate, we kept the questionnaire as brief as possible.

*Demographic characteristics* included age, country of origin, marital status, number of children, and level of education.

*Weight history* was obtained from self-reported height and weight (current, highest, lowest since adulthood, and desired). From these data, body mass indices (BMI) were calculated.

*Aspects of sports activity* were assessed by two questions: 1) “What is the frequency of your sports activity?” (5-point Likert response option ranging from daily to never) (never—for those who only use the spa area), and 2) “Why do you regularly do sports?” offering four options to check with yes or no (e.g., to lose weight, to gain muscularity, to relax, to do competitions).

There are no existing, standardized questions to prove the criteria of “excessive sports” [19] described in DSM-5 as a specific purging method of bulimia nervosa defined by a) “colliding with other important activities or” b) “performed at inappropriate times and places or” c) “continued despite injuries or medical complications” [5, page 546]. Therefore, we used selected items from the Exercise Dependence Scale (EDS-21) [20, 21] and the Exercise Addiction Inventory (EAI) [22, 23] since the whole instruments assessed subscales that were not of interest for our analysis. The instruments aim to assess maladaptive patterns of exercising based on 21 and 6, respectively, items to describe subscales such as “tolerance”, “withdrawal”, “intention effect”, “lack of control” “time”, “reduction in other activities”, “continuance” with six-point Likert response options including 1 = never till 6 = always. The selection of the single items was based on the best matches with the three DSM-5 criteria described above: “I have conflicts with my family or friends because of the amount of my exercise” (= item 2 of the EAI for criteria a); “I think about exercise when I should be concentrating on school or work “ (= item 12 of the EDS) for criteria b); “I exercise despite recurring or persistent physical problems or injuries” (summary of items 2, 9, 16 of EDS for criteria c). At least one of these selected items had to be scored equal or higher than 4 (= often/ very often/ always) in order to meet criteria for “excessive exercising” as described above.

*Eating behavior* was assessed by the self-report Eating Disorder Examination Questionnaire (EDE-Q) [24, 25]. This psychometrically validated instrument describes eating behavior and body image occurring during the past 28 days based on four subscale scores [describing (1) restraint eating, (2) eating concern, (3) shape concern, (4) weight concern] and a global score for these behaviors. We used the men-specific cutoff-score > 1.68 [26] and the common cutoff-score ≥ 2.3 [27] to indicate disturbed or disordered eating.

In addition, in line with prior research [28], we assessed key symptoms of clinical eating disorders as defined by DSM 5 such as (a) BMI < 18.5 plus weight phobia (of anorexia nervosa, AN), (b) binge eating at least once per week for more than 3 months (of binge eating disorder, BED), (c) binge eating and purging (of bulimia nervosa, BN) and (d) purging without binge eating (of purging disorder, PD). Specifically, we classified men as having eating disorder symptoms (EDS) if they reported any of the four *current* characteristics (a, b, c or d). Purging was defined to include vomiting, laxative abuse, diuretic abuse, strict dieting or excessive exercise as methods of weight control. As defined above, we proved criteria of “excessive exercising” as a bulimic purging method according to DSM-5 criteria. Finally, we asked respondents, deliberately low threshold (without criteria of treatment or diagnosis), whether they ever had an eating disorder to also examine subjective awareness of disordered eating. Further, our questionnaire included frequency of lifetime diet behavior to prevent weight gain or to lose weight offering three answering options (never, up till 20 times, and > 20 times).

*Body image* focusing on satisfaction with weight, shape and body was assessed by the three questions: “How satisfied are you with your current weight?”, “How satisfied are you with your current shape?” offering three answers (satisfied, moderate satisfied, dissatisfied) and “Do you like your body?” offering answering options: “yes”, “no”, “I do not know.”

### Data analytic plan

Statistical analysis was performed using SPSS (version 26) [29]. To compare the three age groups based on 305 men (18–40, 41–60 and 61–80 years) with respect to demographic, weight and sport characteristics, we used the Kruskal–Wallis test for ordinal and for continuous variables (as the majority of them was not normally distributed), and the Chi-square test for categorical variables. Post hoc pairwise group comparisons were performed using the Mann–Whitney *U* test and the Fisher’s exact test. As the three groups differed significantly with regard to current BMI, the potential confounding effect of this variable was taken into account in all subsequent analyses. We used analyses of covariance for group comparisons involving continuous variables, logistic regression for binary-dependent variables, and ordinal regression for ordinal-dependent variables, always with adjustment for current BMI. Post hoc pairwise group comparisons were performed only if the overall comparison of the three groups yielded statistical significance (*p* < 0.05). This sequential testing procedure grants in the case of the three groups that the family-wise alpha level of 0.05 is retained without correction for multiple testing [30]. An alpha level of 0.05 was used to determine the statistical significance of all results.

### Power analysis (post hoc sensitivity analysis)

The following power analysis was conducted using the program GPower, version 3.1.7. It is based on standard assumptions regarding type 1 error (alpha = 0.05) and power (1-beta = 0.8). Then the sample of 305 men, split into three age groups of unequal size (age 18–40: *n* = 169, age 41–60: *n* = 81, age 61–80: *n* = 55, see “Results”), is sufficiently large to detect in an analysis of covariance with three groups and up to three covariates, an effect size f = 0.231. This is a medium effect size according to Cohen’s classification [31]. In a logistic regression analysis, e.g., for comparing the three age groups regarding the prevalence of EDS, the above sample size allows detection of ORs of 3.00 for comparison of the two younger age groups and ORs of 3.55 and 3.90 for the other two comparisons (youngest vs oldest group, the two oldest groups), provided that the prevalences to be compared are greater than 0.1. For smaller prevalences, the values of the detectable ORs increase; for rates between 0.05 and 0.1, they are 4.16, 5.14 and 5.74, respectively. These are medium (OR ≈ 2.7 – 4.7) to large (OR > 4.7) effect sizes [32].

## Results

### Demographic, weight and sport characteristics

Table 1 shows a comparison of demographic, weight and sport characteristics by age group. The majority of the demographic and weight-related variables differed significantly between age groups, reflecting their variability over the lifespan. The older the men, the more often they reported to be married or in partnership, to have children, to be less educated or to have higher body mass indices. Current weight categories differed significantly in the three age groups, with 66% of the youngest and 54% of the middle-aged men reporting a BMI in the normal range of 18.5–24.9 as opposed to only 27% of the older men who were highly overweight and obese (73%). Over three-quarters of all men reported daily or intensive sports activity (4–6 times a week), the youngest being significantly more active than the middle-aged and at a trend level than the oldest group. Excessive exercise exhibited significant differences between the groups (46% in youngest versus 22% in middle-aged versus 11% in oldest men), indicating high levels of maladaptive sports behavior. Reasons for sports activity did not distinguish the groups significantly.

**Table 1:** Demographics, weight and sport characteristics

	Total	Age groups			Significance of differences			
		(1) 18–40	(2) 41–60	(3) 61–80	*p*-value	1 vs. 2	1 vs. 3	2 vs. 3
	*N* = 307	*N* = 169(55%)	*N* = 81 (27%)	*N* = 55 (18%)	Overall^a^			
*Demographics*								
Age, M (SD)	40.3 (17.7)	26.3 (5.4)	49.9 (5.4)	69.4 (4.9)	< .001	< .001	< .001	< .001
Austrian origin, *N* (%)	263 (87)	140 (83)	73 (91)	50 (91)	.234			
Married or in partnership, *N* (%)	201 (66)	89 (53)	62 (77)	50 (91)	< .001	< .001	< .001	.039
Education > 12 years, *N* (%)	204 (67)	132 (79)	49 (61)	23 (42)	< .001	.004	< .001	.037
One or more child/ren, *N* (%)	107 (37)	17 (10)	50 (64)	40 (85)	< .001	< .001	< .001	.013
*BMI*^*c*^* current, M (SD)*	25.1 (3.0)	24.4 (2.8)	25.4 (2.8)	26.9 (3.2)	< .001	.009	< .001	.004
BMI lowest since adulthood	22.1 (2.7)	21.7 (2.5)	22.0 (3.1)	23.2 (2.1)	< .001	.202	< .001	.003
BMI highest	27.1 (4.6)	26.3 (4.6)	27.3 (4.7)	29.3 (3.8)	< .001	.006	< .001	.006
BMI desired	24.7 (2.5)	24.5 (2.6)	24.5 (2.3)	25.6 (2.3)	.004	.858	.002	.005
*Current weight categories, N *(%)					< .001	.308	< .001	.011
Underweight (BMI < 18.5)	1 (1)	1 (1)	0	0				
Normal weight range (18.5–24.9)	171 (56)	112 (66)	44 (54)	15 (27)				
Overweight (25.0–29.9)	119 (39)	51 (30)	34 (42)	34 (63)				
Obese (BMI > 30)	12 (4)	4 (2)	3 (4)	5 (10)				
*Sports activity frequency, N* (%)								
Daily	30 (10)	25 (15)	2 (3)	3 (6)	.021	.013	.081	.582
6–4 times/week	200 (66)	111 (66)	55 (68)	34 (62)				
3–1 times/week	72 (24)	30 (18)	24 (30)	18 (33)				
1–3 times/months	2 (1)	2 (1)	0	0				
Less than 1–3 times/months	1 (1)	1 (1)	0	0				
*Excessive exercise* (DSM-5^d^), *N* (%)	102 (33)	78 (46)	18 (22)	6 (11)	< .001	< .001	< .001	.011
*Sports activity in order to, N* (%)								
- Lose weight	83 (43)	49 (39)	21 (50)	13 (50)	.328			
- Gain muscularity	106 (46)	71 (50)	23 (43)	12 (35)	.255			
- Relax	136 (45)	83 (49)	32 (40)	21 (38)	.206			
- Do competitions	71 (23)	37 (22)	19 (24)	15 (27)	.714			

Due to missing observations, frequencies in the individual age groups do not always add up to the total number in this group

^a^Group comparisons by the Kruskal-Wallis test for ordinal and for continuous variables, and the Chi-square test for categorical variables

^b^Adjusted for current BMI by analysis of covariance or ordinal logistic regression (for categorical variables)

^c^Body mass index

^d^Excessive exercise defined by (a) sports activity despite current physical pain or injury or (b) thinking of sports instead of concentrating on work or (c) conflicts with family or friend because of sports activity

### Eating behavior and body image perception

The Global score of the EDE-Q distinguished the three groups significantly, showing a lower score in the middle-aged men compared to both the youngest and the older men (Table 2). Most of the subscales of the EDE-Q revealed similar scores among the three groups, and only the “restraint” scale showed differences. Using the EDE-Q cutoff-scores as indicators for disordered eating, both the general (prevalence rate: 4–6%) and the male-specific cutoff (rate: 9–15%) did not distinguish the three groups. From the perspective of eating disorder symptomatology, 31 (10%) of all male respondents met criteria for current eating disorder symptoms (EDS) most with regard to binge eating, followed by binge eating and purging and purging without binge eating (see Table 2). The three age groups differed significantly from each other by a striking decrease, showing the highest prevalence rates of EDS in the youngest group (14%) and lowest in the oldest group (2%) (adjustment for current BMI did not alter this finding). Of note, purging in the context of bulimic behavior was predominately based on excessive exercising and not on self-induced vomiting as typically associated with pathological eating behavior in women. Lifetime restricting dieting indicated no differences between the groups and prevalence rates were low in males of all groups. The question with regard to lifetime eating disorder resulted in three positive answers in the whole sample (2%).

**Table 2:** Eating behavior and body image

	Total	Age groups			Significance of differences				*p*-value
		18–40	41–60	61–80	*p*-value	1 vs. 2	1 vs. 3	2 vs. 3	
	*N* = 307	*N* = 169 (55%)	*N* = 81 (27%)	*N* = 55 (18%)	Overall^a^				Adjusted^b^
*EDE-Q*^*c*^*, Global Score, M (SD)*	0.8 (0.7)	0.9 (0.7)	0.7 (0.7)	0.9 (0.8)	.039	.022	.635	.033	.065
- Restraint	1.2 (1.2)	1.3 (1.2)	1.0 (1.2)	1.1 (1.4)	.028	.012	.125	.520	.010
- Eating concern	0.3 (0.5)	0.3 (0.5)	0.2 (0.5)	0.3 (0.5)	.488				
- Weight concern	0.8 (0.9)	0.8 (0.9)	0.7 (0.9)	1.0 (1.0)	.089				
- Shape concern	1.0 (1.1)	1.0 (1.0)	0.9 (1.1)	1.2 (1.1)	.214				
EDE-Q cutoff > 1.68, *N* (%)	34 (11)	18 (11)	7 (9)	8 (15)	.562				
EDE-Q cutoff > 2.3, *N* (%)	15 (5)	8 (5)	5 (6)	2 (4)	.752				
*Any eating disorder symptoms (EDS), N* (%)	31 (10)	24 (14)	6 (7)	1 (2)	.019	.148	.012	.241	.035
*EDS*^*d*^* type, N* (%)					.188				
- BMI < 18.5 and fat phobia	0	0	0	0					
- Binges eating	23 (8)	17 (10)	5 (6)	1 (2)					
- Binges and purging*	5 (2)	4 (2)	1 (1)	0					
*Vomiting*	*1*	*1*	*0*	*0*					
*Excessive exercise*	*4*	*3*	*1*	*0*					
- Purging behavior	3 (1)	3 (2)	0	0					
*Vomiting*	*3*	*3*	*0*	*0*					
- No symptom	274 (90)	145 (86)	75 (93)	54 (98)					
*Lifetime restricting diet behavior, N* (%)					.672				
Never	185 (61)	102 (61)	51 (64)	32 (59)					
Up till 20 times	104 (34)	61 (36)	24 (30)	19 (35)					
> 20 times	13 (4)	5 (3)	5 (6)	3 (6)					
*Lifetime history of an eating disorder, N* (%)	3 (2)	3 (3)	0	0	.495				
*Body image*									
*Satisfaction with weight? N* (%)					.001	.003	.003	.220	.167
Satisfied	193 (64)	103 (61)	58 (73)	32 (58)					
Moderately satisfied	73 (24)	53 (31)	10 (13)	10 (18)					
Dissatisfied	38 (13)	13 (8)	12 (15)	13 (24)					
Satisfaction with shape? *N* (%)					.006	.002	.175	.122	.006
Satisfied	185 (61)	94 (56)	59 (75)	32 (58)					
Moderately satisfied	85 (28)	60 (36)	11 (14)	14 (26)					
Dissatisfied	33 (11)	15 (9)	9 (11)	9 (16)					
"*Do you like your body? N* (%)					.049	.126	.170	.008	.094
Yes	244 (81)	136 (81)	70 (89)	38 (69)					
No	48 (16)	25 (15)	9 (11)	14 (26)					
Do not know	10 (3)	7 (4)	0	3 (6)					

Due to missing observations, frequencies in the individual age groups do not always add up to the total number in this group

^a^Group comparisons by the Kruskal–Wallis test for ordinal and for continuous variables, and the Chi-square test for categorical variables

^b^Adjusted for current BMI by analysis of covariance or ordinal logistic regression (for categorical variables)

^c^EDE-Q = Eating Disorder Examination Questionnaire

^d^EDS = Eating disorder symptoms

With regard to body image, the majority of all men were satisfied with their weight and shape and agreed to the sentence: “I like my body”. However, comparison by age group revealed significant differences between all three groups showing the middle-aged men as the most satisfied with weight and shape and most body liking group compared to the youngest and the oldest, respectively. However, after adjusting for current BMI, only satisfaction with shape remained significant.

## Discussion

We used an anonymous questionnaire to assess eating behavior including prevalence rates of eating disorder symptoms, frequency and intensity of sports activity, and body image in 307 fitness center-attending men, aged 18–80 years, recruited from two of the largest fitness centers in Innsbruck, Austria. To the best of our knowledge, this is the first study of fitness center men on eating behavior and associated features. Our study yielded four main findings.

First, we found disturbed eating behavior in all age groups assessed by the EDE-Q global score and based on both EDE-Q cutoff scores (general and men specific as described above). Our findings in fitness center men differ from a prior community study [33] that included women and men, aged 24 up till 75 years based on the comparison with the data presented in the graph of Hilbert et al.’s study [33]. Our sample seems to show much higher scores (almost double) of the EDE-Q (with the exception of the “eating concern”), indicating pathological eating behavior as compared to the male subjects of the Hilbert study (values were drawn from the graph and compared to ours). In line, the fitness center men of our study displayed more than three times higher rates of the EDE-Q cutoff score ≥ 2.3 as compared to the community men (5% vs 1.5%). In future studies, a statistical-based comparison should be performed.

Secondly, with regard to eating disorder symptomatology, 10% of all men met criteria with the highest rates observed in the youngest group, followed by the middle-aged and the oldest men. Binge eating was the most frequent symptom, followed by binging and purging. Men with bulimic symptoms predominately reported using excessive exercise to compensate for binging. This finding goes along with results from an earlier study of our group on eating disorder symptoms in community men aged 40–75 years [18]. In this sample (*N* = 470), we found 7% meeting criteria for eating disorder symptoms as described above and four out of seven who used excessive exercising (maladaptive pattern) as the purging method. Similar to “muscularity-oriented eating disorders” as described recently [11, 12], excessive exercising appears as a male-specific characteristic in the context of eating disorders. Excessive exercising, previously described as an expression of male coping strategies for comorbid depression [34], may serve in the context of disordered eating as a strategy for acute emotion regulation [35, 36].

Thirdly, our data show a large discrepancy between eating disturbances as identified by EDE-Q and DSM-5 symptoms and the self-reported eating disorders suggesting a lack of awareness of the pathological pattern of eating behavior in men. Only 3 men reported a lifetime eating disorder, while we found 31 men with EDS and 15–34 men with disordered eating as defined by EDE-Q cutoff scores. This could be due to the fact that eating disorders are still predominately associated with female gender, and therefore pathological eating behavior especially when related to sports activity is just not associated with “eating disorders” by concerned men [37].

Fourthly, variables on body image showed high proportions of men who are satisfied with their weight, shape, and their body as a whole. This differs strongly from studies on women across ages [e.g. 38].The intensity of their training (almost 80% of all men exercised 4–7 times per week) could be one reason for the high proportion of positive body image, as well as the consequence of the high score of restraint eating (= high discipline in eating behavior including healthy and low caloric nutrition).

### Strengths and limits

This study adds to the very limited data on eating behavior in men of different ages in the context of fitness sport.

Several limitations of the study should be recognized. First, our participation rate of this study was 46% raising the possibility of non-response bias and selection bias, respectively. However, our response rate is equivalent to the finding of a meta-analysis of Shih and Fan [39] who showed response rates of mail surveys to be 10% higher than the rates of web surveys (45% vs. 34%). Further, since eating disorders are often secret, participants with disordered eating may have been less likely to respond than those without, causing an underestimation of the true rate. On the other side, the true prevalence of eating behaviors could be overestimated such as binge eating if individuals with this behavior were more likely to respond or if they overstated the severity. Also, the combination of disordered eating and excessive exercise might be associated with hyperactivity and/or impulsivity, characteristics that could cause rejection of study participation. Second, the classification into weight categories based on current BMI could be biased due to the lack of assessment of body fat or muscularity. However, most people do not know their exact proportion of body fat, and therefore we did not include this question in our questionnaire. This should be considered in future studies. Thirdly, we used the EDE-Q next to other variables. It is important to state that the EDE-Q as a questionnaire is not able to assess the complexity and multidimensionality of "eating behavior" and "body image. Fourthly, we used a selection of items of the EDS-21 and the EAI to prove the criteria of “excessive sports” described in DSM-5. This has to be considered carefully in future studies. Finally, we used self-report eating disorder questions and key symptoms of eating disorder diagnosis, rather than performing clinical interviews. Therefore, it is important to state that our findings are indicative of disordered eating. However, our questions were based on standardized instruments (e.g., the EDE-Q and the DSM-5 clinical criteria for eating disorders) which are commonly used in research on eating behavior [28] and in our prior studies in men [40].

In summary, the present results provide clear evidence that disordered eating based on EDE-Q and DSM-5 eating disorder symptoms do occur in men across all ages attending fitness centers. In addition, our findings indicate that excessive exercise is a male-specific purging method after binging. Furthermore, it appears that in the context of sports activity eating disorder symptoms are mostly under-recognized by affected men, possibly due to the fact that eating disorders are still seen as primarily female disorders. More male-specific research is needed to increase attention and knowledge about disordered eating in men of fitness centers and its special characteristics.

## What is already known on this subject?

Studies on men are still less than 1% of all eating disorder research. There are special characteristics of disordered eating in men such as the focus on muscularity and sports [11, 37]. Including these aspects, the prevalence numbers has risen up to 20–30% of risky eating behaviors [37].

## What does this study add?

We compared younger, middle-aged and older men and found disordered eating as defined by the EDE-Q and DSM-5 key symptoms (i.e., BMI < 18.5 and fat phobia, binge eating, purging behaviors, excessive exercise) in all groups. Our results showed that 10% of fitness-center attendees had symptoms of disordered eating and that the sport context may be involved with this [38].

## Data Availability

The data that support the findings of this study are available from the corresponding author upon reasonable request.
